# Supplementary material for: Using fluorescence flow cytometry data for single-cell gene expression analysis in bacteria
Source: PLoS One. 2020 Oct 12;15(10):e0240233. doi: 10.1371/journal.pone.0240233 (PMC7549788; doi:10.1371/journal.pone.0240233)
Supplement: S1 File — (PDF) [file pone.0240233.s001.pdf]

Supplementary Materials for "Using fluorescence flow cytometry data for single-cell gene expression analysis in bacteria"

Luca Galbusera[1,2], Gwendoline Bellement-Theroue[1,2], Arantxa Urchueguia[1,2], Thomas Julou[1,2*], Erik van Nimwegen[1,2*]

1 Biozentrum, University of Basel, Basel, Switzerland
2 Swiss Institute of Bioinformatics, Lausanne, Switzerland

* thomas.julou@unibas.ch, erik.vannimwegen@unibas.ch

1 Flow cytometers signals and their statistical properties

1.1 Signal acquisition

In the flow cytometer, each cell is made to pass through a set of laser beams in order to measure its scattering profile and its fluorescence intensity at several wavelengths. The scatter has two components, forward- and side-scatter, which are usually interpreted as follows [1]:

1. Forward-scatter (FSC) generally reflects the size of the cells or particles.
2. Side-scatter (SSC) generally reflects the internal complexity or granularity of the cells or particles.

The scatter and fluorescence of each event (typically a cell) is converted by a photomultiplier tube into a pulse of electrical signal that is characterized by a height and a duration (Fig. 1 of main text). Roughly speaking, a pulse begins when a particle enters the laser beam and it reaches its maximum when the particle reaches the middle of the beam. In order to represent the pulse, the signal is sent to an analog-to-digital converter (ADC) that discretizes the continuous pulse into a digital one. For the machine used in this study, the ADC does this by sampling the pulse every 0.1 μsec and the height of the signal is mapped to an integer between 0 and 2^{14}, i.e. 14 bits are used to quantify the height of the signal [2].

1.2 Correlations among the signals

The cytometer gives three statistics of a measured pulse (height, width, and area), and we investigated correlations between these statistics. For the machine used in our study, we find that the area is in fact exactly proportional to the product of the height and width of the signal (Suppl. Fig. S1).

Consequently, of the three statistics reported, only two are independent and we next measured the correlations between all pairs of statistics to determine which of these are most independent. As shown in Suppl. Fig. S2 we can see that area correlates significantly with both height and width whereas height and width do not show significant correlation. We thus decided to characterize each signal by height and width.

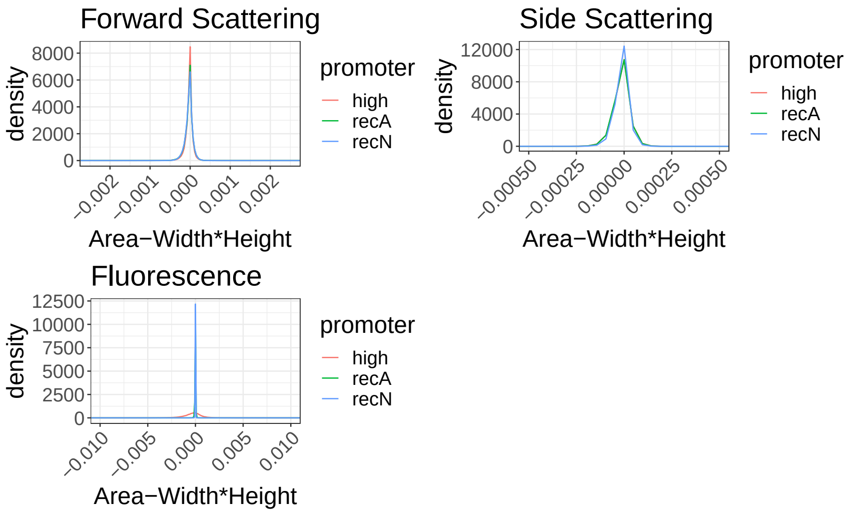

Fig S1. Pulse area is proportional to the product of height and width. Apart from very rarely occurring negative or saturated signals, the reported area of each pulse is equal to a constant C times the product of the reported height and width. The histograms show the distribution of the difference area $- C \cdot$ width $\cdot$ height, which is highly concentrated around zero. Different colors represent different promoters.

1.3 Signal width is not informative for fluorescence measurements

To assess the relative importance of the height and width statistics for fluorescence measurements we made use of the calibration beads manufactured by BD, which come in a set of three different expression levels. Supplementary Fig. S3 shows that, whereas the heights of the signal pulses show three main peaks, corresponding to the three intensities of the beads, the distribution of measured widths is almost identical for all beads. Consequently, we chose to only use signal height to quantify fluorescence.

1.4 Anomalous behavior at low fluorescence intensities

As can already be observed from the measurements of the calibration beads (Suppl. Fig. S3), due to shot noise in the fluorescence measurements, the coefficient of variation generally increases as the fluorescence level decreases. This general negative correlation between mean fluorescence and its coefficient of variation can not only be observed for the calibration beads, but also for a library of transcriptional reporters of native *E. coli* promoters (Suppl. Fig. S4). However, as Suppl. Fig. S4 also shows, when fluorescence levels become very low, the CV^2 of the measurements reaches a maximum and starts to *decrease* as the mean decreases further, and the lowest CV^2 is observed for cells without any GFP expression. We hypothesize that this decrease in CV^2 for very low fluorescence levels derives from the fact that autofluorescence levels vary less than GFP fluorescence levels, in combination with some specific signal processing that is performed by the machine at low signal intensities. Although we invested a significant amount of time in trying to reverse engineer what signal processing may cause this anomalous behavior at very low fluorescence levels, these attempts were ultimately not successful. In addition, our repeated requests to BD to bring us into contact with the relevant technicians went unanswered. Consequently, we were forced to restrict our quantitative analysis to cells whose GFP levels were at least twice the autofluorescence level (corresponding the log-mean levels roughly equal to those of the dimmest

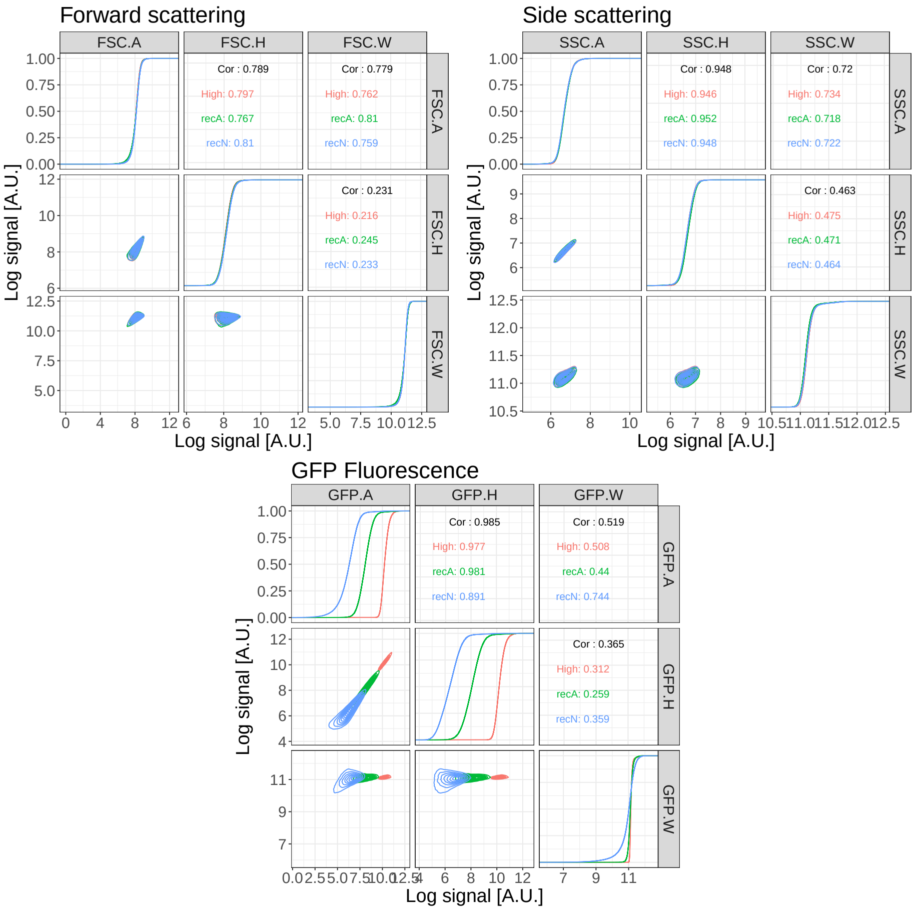

Fig S2. Correlations among the different statistics reported for each signal pulse. The plots show the correlations among the height (H), area (A) and width (W) of the signal pulses for the forward- (FSC) and side-scatter (SSC) as well as the fluorescence measurements (GFP). The colors represent 3 different promoters, two native promoters (recA and recN) and one synthetic promoters (high).

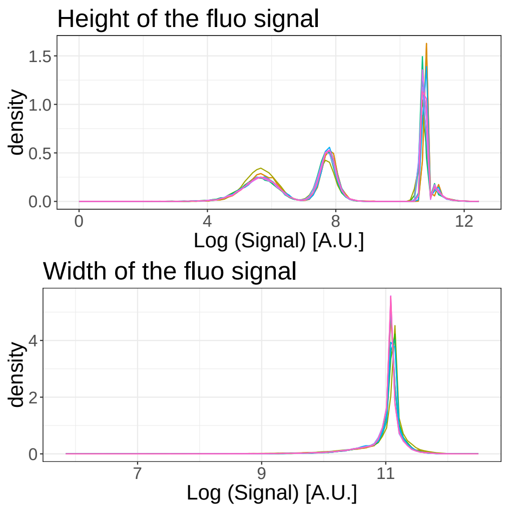

Fig S3. Population separation of calibration beads based on their fluorescence signal pulses. Distributions of the measured heights (top) and widths (bottom) of the fluorescence signal pulses for populations of artificial beads. Each color corresponds to one measurement run of a set of artificial beads consisting of a mixture of beads of three different fluorescent intensities. We see that, whereas signal height shows 3 clearly separated peaks, the distribution of signal widths is narrowly peaked at a single value. Note also the notably wider variance of the peaks in fluorescence height at low intensities than at high intensities, which results from measurement shot noise.

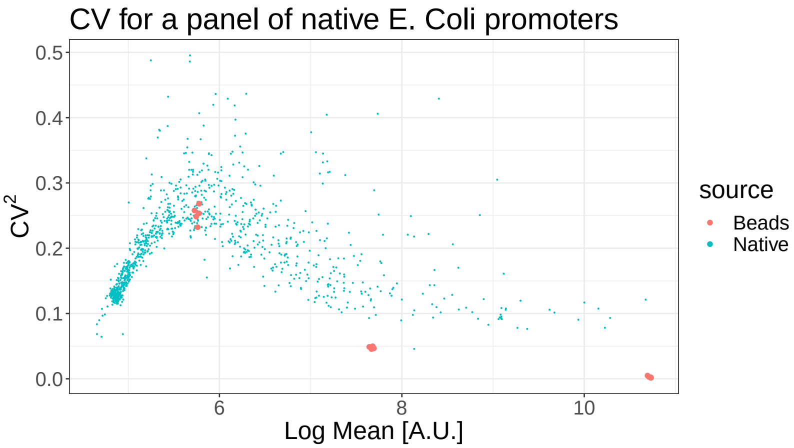

Fig S4. Coefficient of variation as a function of mean fluorescence level exhibits anomalous behavior at low fluorescence. The blue scatter shows the measured coefficient of variation squared CV^2 (vertical axis) as a function of the logarithm of mean fluorescence level (horizontal axis) for a library of transcriptional reporters of native *E. coli* promoters [3] as obtained in a recent study from our lab [4], with each blue point corresponding to one promoter of the library. The red points show the same statistics for the calibration beads at three intensities. Note that, due to shot noise in the fluorescence measurements, the CV^2 increases as the mean fluorescence decreases, including for the calibration beads (that have low intrinsic variance). However, at very low fluorescence, the CV^2 reaches a maximum and decreases for even lower fluorescence.

calibration beads in Suppl. Fig. S4).

2 Mixture model of the scatter measurements

The scatter of each measured event is characterized by 4 statistics: the height and width of both the forward- and side-scatter. We fit the set of 4D scatter measurements for a given dataset by a mixture of a multivariate Gaussian and a uniform distribution. The idea is that the Gaussian distribution models the viable cells, while the uniform distribution represents outliers that may correspond to fragments of dead cells or other debris. Concretely, we assume that the probability to observe the 4D vector $\vec{y}$ for a single measured event is given by

$$P(\vec{y}\,|\,\vec{\mu},\Sigma,\rho) = \frac{\rho}{\sqrt{(2\pi)^4|\Sigma|}} e^{-\frac{1}{2}(\vec{y}-\vec{\mu})^T\Sigma^{-1}(\vec{y}-\vec{\mu})} + \frac{1-\rho}{\Delta}, \tag{1}$$

where $\vec{\mu}$ is the center of the 4D multivariate Gaussian, Σ its covariance matrix, ρ the fraction of viable cell measurements in the dataset, and Δ is the volume of the 4D hypercube spanned by all the data points. Note that $|\Sigma|$ denotes the determinant of the covariance matrix. The *E-Flow* package that we distribute contains a C++ implementation where the model (1) is fit to a given dataset using expectation maximization. An example of the results of this fitting to a dataset of *E. coli* cells grown in minimal media with lactose is shown in Fig. 2 of the main text, showing that the observed 4D scatter can be well approximated by the mixture model.

Once the mixture model is fitted, we can calculate a posterior probability p_i for each observation i that it derives from the Gaussian mixture component, i.e. that it is a viable cell measurement. By default *E-Flow* filters out all events i for which this posterior probability is less than 0.5 and retains all measurements with posterior probability larger than 0.5, but users can choose to alter this threshold in posterior probability. Supplementary Figure S5 shows the same 4D scatter of measurements as shown in Fig. 2 of the main text, but now with all selected events in red, and all events that were filtered out in black.

To investigate the effect of the filtering strategy on the observed fluorescence levels we calculated the distribution of fluorescence levels of several reporters when using either a very strict threshold, retaining only cells with posterior $p > 1 - e^{-10}$ or a very lenient threshold, retaining all cells with $p > e^{-10}$. As shown in Suppl. Fig. S6 there is virtually no difference between the observed distribution of fluorescence with the two thresholds. This observation strongly suggests that it is not possible to effectively pick out a subpopulation of cells of similar size by strict gating on forward- and side-scatter.

3 Inferring the mean and variance of the distributions of fluorescence levels

After filtering events based on forward- and side-scatter, we next fit the distribution of fluorescence by a mixture of a log-normal distribution, which captures valid measurements and corresponds to the bulk of the measurements, and a uniform distribution capturing outliers. In particular, we assume that the probability for a fluorescence measurement to have log-fluorescence x is given by the mixture:

$$P(x|\rho,\mu,v) = \frac{\rho}{\sqrt{2\pi v}} \exp\left[-\frac{(x-\mu)^2}{2v}\right] + \frac{1-\rho}{\Delta}, \tag{2}$$

where μ and v are the mean and variance of log-fluorescence levels, ρ is the fraction of 'valid' measurements and $\Delta = x_{\text{max}} - x_{\text{mix}}$ is the observed range of log-fluorescence

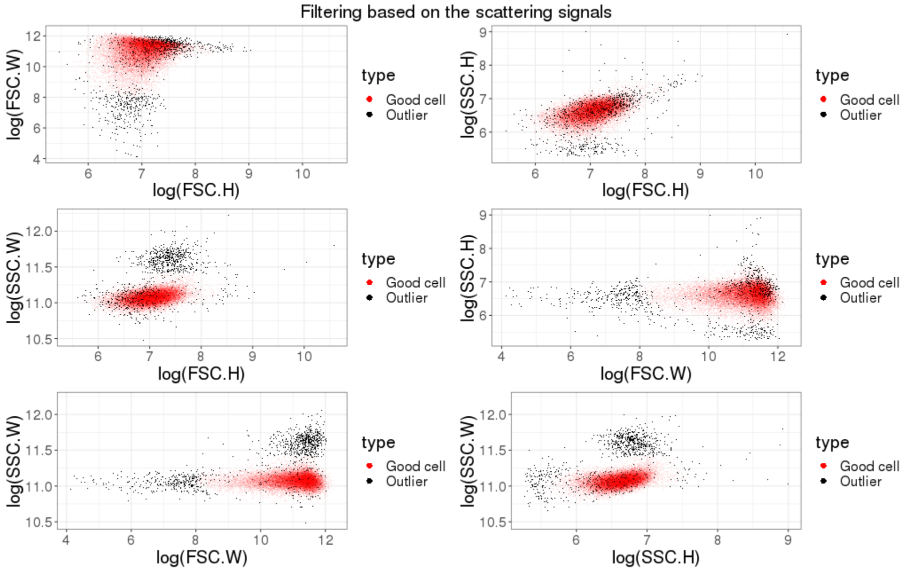

Fig S5. Filtering of events based on their scattering profiles. The panels show a scatter of the forward- and side-scatter measurements for a set of *E. coli* cells grown in minimal media with lactose. Events that have posterior probability larger than 0.5 to stem from the Gaussian component of the mixture are indicated in red, and events with posterior less than 0.5 are superimposed in black. The red events are considered viable cell measurements and the black events are discarded. Note that around 4% of the measured events are discarded for this dataset.

levels. The log-likelihood for a dataset of n measurements x_i is then simply given by

$$L(\rho,\mu,v)=\sum_i \log\left[P(x_i|\rho,\mu,v)\right]. \tag{3}$$

We maximize the log-likelihood (3) using expectation maximization to obtain the fitted parameters ρ_*, μ_* and v_*. To obtain error bars σ_μ and σ_v on the mean μ and variance v we obtain the Hessian matrix of the log-likelihood by expanding to second order around its maximum and we take the diagonal elements of its inverse. For any given set of fluorescence measurements, our package *E-Flow* returns both the fitted values (μ_*, v_*) and their error bars (σ_μ, σ_v).

Below we develop methods for correcting the observed fluorescence levels for both cell autofluorescence and for the shot noise in the FCM measurements. In order to do this we also need the mean and variance of fluorescence levels, rather than just the mean and variance of *log*-fluorescence levels. That is, if we write for the fluorescence of a cell $f=e^x$, we need to obtain the mean and variance of f. Using the well-known expressions of the mean and variance of a log-normal distribution we have

$$\langle f\rangle = e^{\mu_*+v_*/2}, \tag{4}$$

and

$$\mathrm{var}(f)=\left[e^{v_*}-1\right]e^{2\mu_*+v_*}. \tag{5}$$

Further, given that the error bars on the mean and variance are generally small compared to their means, we use a linear approximation to calculate error bars on the mean and variance of f, and find

$$\sigma_{\langle f\rangle}=\langle f\rangle\sqrt{\sigma_\mu^2+\frac{\sigma_v^2}{4}}, \tag{6}$$

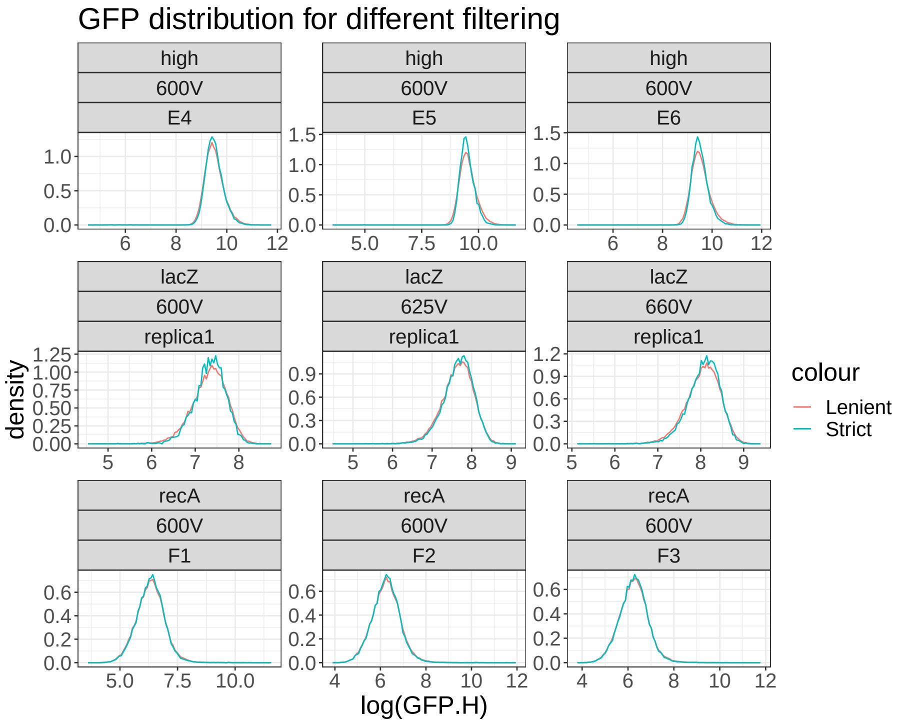

Fig S6. Fluorescence distribution when using very strict or very lenient gating of forward- and side-scatter. Each panel shows the observed distribution of fluorescence levels of a transcriptional reporter (rows) using different replicate measurements (columns) when using either a very strict threshold $p > 1 - e^{-10}$ or a very lenient threshold $p > e^{-10}$ for filtering events. Note that the name of the reporter and the voltage of the flow cytometer's laser used in a given replicate are indicated at the top of each panel.

and

$$\sigma_{\mathrm{var}(f)} = \sqrt{4\mathrm{var}(f)^2\sigma_\mu^2 + \left(2\mathrm{var}(f) + \langle f\rangle^2\right)^2\sigma_v^2}. \tag{7}$$

The *E-Flow* package also returns these estimates and error bars for fluorescence levels f.

4 Averaging of replicate autofluorescence measurements

As shown in Fig. 4 of the main paper, we measured the autofluorescence distributions of cells that do not express GFP in triplicate on 4 separate days, and for two different strains. Noticing that there appears to be a small but systematic variation in both the means and variances of the autofluorescence levels, we proceeded as follows to estimate an overall average for the mean and variance of autofluorescence, adapting a method first presented in [5].

We assume that there is an overall mean autofluorescence μ and that on any given day d, the mean autofluorescence μ_d on that day deviates from μ by some amount δ_d, i.e.

$$\mu_d = \mu + \delta_d. \tag{8}$$

We will assume that the deviation δ_d varies by an (unknown) amount τ across days, following a Gaussian distribution. That is, the probability of having mean autofluorescence μ_d on any given day is

$$P(\mu_d|\mu,\tau) = \frac{1}{\sqrt{2\pi}\tau}\exp\left[-\frac{(\mu_d-\mu)^2}{2\tau^2}\right]. \tag{9}$$

Let μ_{id} be the estimated mean autofluorescence of replicate i on day d, and let σ_{id} be the associated error-bar on this mean. Assuming that the true mean autofluorescence on day d was μ_d, the probability to obtain a measured mean autofluorescence μ_{id} is also given by a Gaussian distribution:

$$P(\mu_{id}|\sigma_{id},\mu_d) = \frac{1}{\sqrt{2\pi}\sigma_{id}}\exp\left[-\frac{(\mu_{id}-\mu_d)^2}{2\sigma_{id}^2}\right]. \tag{10}$$

The probability of the data D, i.e. all replicate measurements μ_{id} given an overall mean μ and variance across days τ^2 is given by taking the product over all measurements and days, and marginalizing over all day-dependent means μ_d:

$$P(D|\mu,\tau) = \prod_d\left[\int d\mu_d P(\mu_d|\mu,\tau)\prod_i P(\mu_{id}|\sigma_{id},\mu_d)\right]. \tag{11}$$

These integrals can all be performed analytically and we obtain

$$P(D|\mu,\tau) = \prod_d \frac{1}{\sqrt{2\pi(\tau^2+\sigma_d^2)}}\exp\left[-\frac{(\bar{\mu}_d-\mu)^2}{2(\tau^2+\sigma_d^2)}\right], \tag{12}$$

where we have defined the measured mean $\bar{\mu}_d$ on day d as

$$\bar{\mu}_d = \frac{\sum_i \mu_{id}/\sigma_{id}^2}{\sum_i 1/\sigma_{id}^2}, \tag{13}$$

and the squared error σ_d^2 on the measured mean on day d as

$$\sigma_d^2 = \left[\sum_i \frac{1}{\sigma_{id}^2}\right]^{-1}. \tag{14}$$

If we define the weighted overall average

$$\bar{\mu} = \frac{\sum_d \bar{\mu}_d/(\tau^2 + \sigma_d^2)}{\sum_d 1/(\tau^2 + \sigma_d^2)}, \tag{15}$$

and the overall squared error

$$\sigma^2 = \left[\sum_d \frac{1}{\tau^2 + \sigma_d^2} \right]^{-1}, \tag{16}$$

then we can rewrite equation (12) as

$$P(D|\mu, \tau) = \left[\prod_d \frac{1}{\sqrt{2\pi(\tau^2 + \sigma_d^2)}} \right] \exp\left[-\frac{(\mu - \bar{\mu})^2}{2\sigma^2} + \frac{\bar{\mu}^2}{2\sigma^2} - \sum_d \frac{\bar{\mu}_d^2}{2(\tau^2 + \sigma_d^2)} \right]. \tag{17}$$

We can then finally marginalize over μ to obtain a likelihood that depends only on τ and the measurements:

$$P(D|\tau) = \sigma \left[\prod_d \frac{1}{\sqrt{2\pi(\tau^2 + \sigma_d^2)}} \right] \exp\left[\frac{\bar{\mu}^2}{2\sigma^2} - \sum_d \frac{\bar{\mu}_d^2}{2(\tau^2 + \sigma_d^2)} \right]. \tag{18}$$

We maximize equation (18) with respect to τ to find the optimal value τ_*. We then substitute this value τ_* into equations (15) and (16) to obtain a final estimate $\bar{\mu}$ of the overall mean autofluorescence and an error bar σ on this estimate. The same procedure is used to estimate average variances $\bar{v}_d$ for each day and an overall average variance $\bar{v}$. The *E-Flow* package returns all these values as the overall estimates of autofluorescence averaged over multiple replicates and days.

5 Fitting the shot noise strength using calibration beads

The electronic cascade in the photomultiplier tube introduces a noise whose variance can be described as a Gaussian with a variance proportional to the square root of the incoming signal [6]. This means that, for every measurement of fluorescence, the relationship between measured intensity I_M and true intensity I_T is given by

$$I_M = I_T + O + \epsilon\sqrt{I_T}, \tag{19}$$

with $\epsilon \sim \mathcal{N}(0, \delta)$ and O is an offset introduced by the electronics in order to avoid negative values when I_T is very small.

To infer the strength δ of the shot noise term we used a set of calibration beads consisting of a mixture of beads of three different expression levels. The top right panel of Suppl. Fig. S7 shows the observed distribution of expression levels for one set of beads showing three main peaks and one small additional peak at very high intensity. We believe that this highest peak is an artifact due to aggregation of multiple beads. We fit the distribution of bead intensities to a mixture of a uniform distribution (to account for outliers) plus four Gaussians. We discard all points assigned to the uniform distribution and the highest Gaussian component, and estimated the means, variances, and CV^2 for each of the remaining three Gaussians. This procedure was repeated for multiple datasets. In total we estimated means, variances, and CV^2 for 31 datasets of calibration beads. The top left panel of Suppl. Fig S7 shows the CV^2 as a function of the mean intensities of the beads across all datasets showing that CV^2 drops approximately inversely with mean expression, as expected for shot noise.

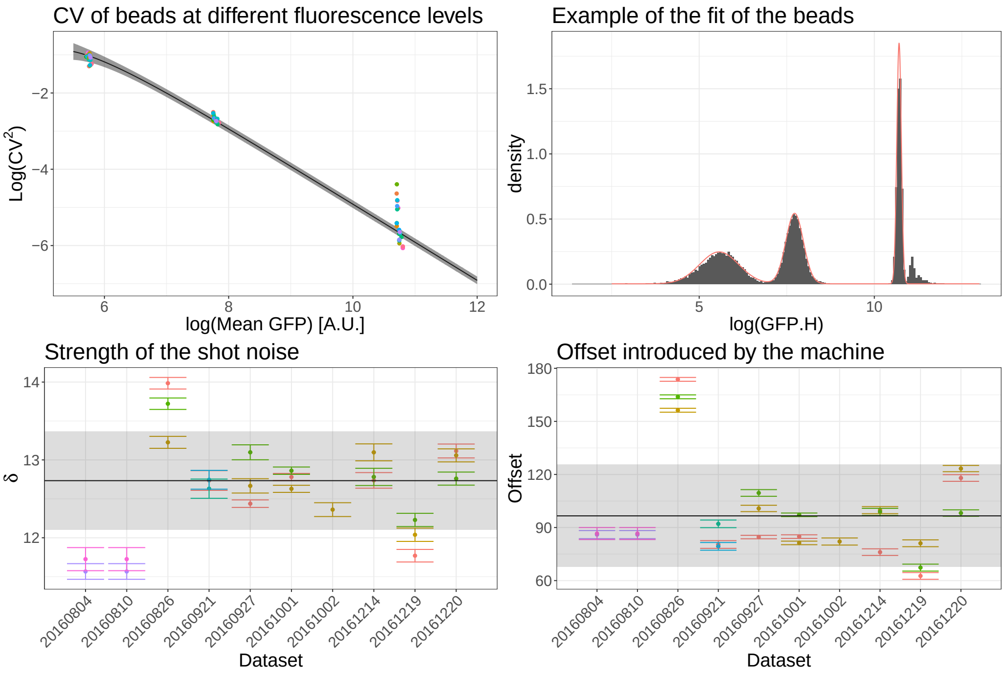

Fig S7. Estimation of the shot noise strength using calibration beads. Top left: CV^2 as a function of mean intensity for each Gaussian component of each mixture of beads analyzed. Both axes are shown on a log-scale and different colors refer to different datasets. The black line and the shaded region represent the fit obtained using a shot noise strength $\delta = 12.7 \pm 0.6$. Top right: Example of the intensity distribution of a single dataset of beads; the red line shows the fit with a mixture of three Gaussians and one uniform. Bottom: Estimates of the strength δ (left) and offset O (right) for different datasets; the estimation is done fitting a model of the form given by equations (24) and (25). The black lines correspond to the means of δ and O and the gray regions correspond to their variation among datasets.

Given one dataset of beads expressions, we can now fit a model based on Eq. (19). First of all it can be proved that under this model the mean and variance of the expression intensities of one single peak i are given by

$$M_M^i = M_T^i + O \tag{20}$$
$$S_M^i = C^2 (M_T^i)^2 + \delta^2 M_T^i \tag{21}$$

where we have supposed that the true coefficient of variation C is the same for every peak (we assume that each of the 3 types of beads has the same manufacturing accuracy in their fluorescence intensities). From the histogram in Fig. S7 (top right) it is clear that in logarithmic space the intensities are well described by Gaussian distributions, which means that M_M^i and S_M^i are the mean and variance of a log-normal distribution. We can work out the μ and σ of a normal distribution in log-space that would have M_M^i and S_M^i as mean and variance in real space

$$s_m^i = \log\left[1 + \left(C \frac{M_T^i}{M_T^i + O}\right)^2 + \delta^2 \frac{M_T^i}{(M_T^i + O)^2}\right] \tag{22}$$

$$\mu_m^i = \log\left(M_T^i + O\right) - \frac{s_m^i}{2} \tag{23}$$

It follows that in logarithmic space the probability of observing a data point x_k (including also a uniform distribution that takes care of possible outliers) is

$$P(x_k \,|\, \vec{M}_T, O, C, \delta, \vec{\rho}) = \sum_{i=1}^{3} \rho_i \mathcal{N}[x_k;\, \mu_m^i(M_T^i, O, C, \delta), s_m^i(M_T^i, O, C, \delta)] + + \frac{1 - \sum_{i=1}^{3} \rho_i}{\Delta} \tag{24}$$

where $\vec{\rho}$ is a vector of weights whose elements sum to one and Δ is the range of the data in logarithmic space. The log-likelihood of the data is

$$\mathcal{L}(\vec{x} \,|\, \vec{M}_T, O, C, \delta, \vec{\rho}) = \sum_{k=1}^{N} \log\left[P\left(x_k \,|\, \vec{M}_T, O, C, \delta, \vec{\rho}\right)\right] \tag{25}$$

This likelihood is maximized using a coordinate descent algorithm and the error bar on δ was estimated by expanding the log-likelihood to second order around its maximum, keeping all other parameters fixed. The same procedure was used to estimate an error bar on the offset O.

After obtaining estimates of δ and O and their error bars for each dataset, we averaged the δs and Os from different datasets in the same way as we did for autofluorescence measurements above. The bottom part of Suppl. Fig. S7 shows the estimates of the shot noise and offset for different datasets and the final average of all these estimates. We find that δ has a value of 12.7 with a variation of 0.6 among datasets. Finally, the black line on the top left panel of Suppl. Fig. S7 shows that the model correctly describes the observed decrease in CV^2 with mean.

6 Mother machine statistics

6.1 Age distribution in the Mother machine

Especially for highly expressed genes the GFP concentration varies relatively little across cells, so that the total amount of GFP in a cell correlates well with its size. Since

each cell expands exponentially along its division cycle in exponential growth, cell size correlates well with the 'age' of a cell, i.e. where it is in its division cycle. For this reason we expect a correlation between the total GFP distribution and the age distribution of the population, i.e. the relative frequencies of cells in different stages of their division cycle. Thus, in order to meaningfully compare GFP distributions from the FCM and from cells growing in a microfluidic device, we must check that the age distributions of the two populations are indeed comparable.

The cell population analyzed in the FCM is expected to have an over-representation of younger cells, since for every old cell that divides two young cells are produced whereas, in the microfluidic device, we expect less over-representation of young cells due to the fact that cells continuously leave the growth channel.

To infer the population structure of a growing population of cells, we use the Leslie approach [7]. We discretize the time in steps of 3 minutes, corresponding to the acquisition time of the time lapse microscopy, and we look for the probability $P(a,t)$ of having cells of age a at time t. The Leslie theory is based on two quantities, the fecundity $f(x,t)$ which represents the expected contribution from an individual aged x at time point t to the population aged 1 at time point $t+1$, and the probability $P(x,t)$ of survival over one time point for individuals who are present in age-class x at time t. These quantities form the Leslie matrix, which is defined as

$$L(t) = \begin{pmatrix} f(1,t) & P(1,t) & 0 & \dots & 0 \\ f(2,t) & 0 & P(2,t) & \dots & 0 \\ \vdots & \vdots & \vdots & \ddots & \vdots \\ f(d-1,t) & 0 & 0 & \dots & P(d-1,t) \\ f(d,t) & 0 & 0 & \dots & 0 \end{pmatrix} \tag{26}$$

this matrix is a squared $d \times d$ matrix where d is an upper limit on the age of the cells, after which $P(x,t) = 0$. The population structure at time t is given by a vector $\vec{n}(t)$ of dimension d, where the entry i is the number of individual in the population of age i. The theory states that

$$\vec{n}(t) = \vec{n}(0) \cdot L^t \tag{27}$$

where we use the convention that vectors are represented by rows. If the matrix L is diagonalizable and its entries are independent of time, it can be shown that for very large t the population reaches a stable age distribution with

$$\vec{n}(t) \sim \lambda_1^t \vec{n}(0) \cdot \vec{U}_1 \vec{U}_1^{-1} \tag{28}$$

$$n_{tot} \propto e^{t \log(\lambda_1)} \tag{29}$$

where λ_1 and $\vec{U}_1$ are the leading eigenvalues and eigenvectors of L.

From the data from the microfluidic device we can estimate the probability for a cell of age x at time t to survive one more time point by computing the probability that the division occurs at an age larger than x; the fecundity is then given as twice the probability that the cell doesn't survive. Suppl. Fig. S8 shows the computed $P(x,t)$ by considering cells at different times t from the beginning of the experiment and it shows that they are all the same, suggesting that the Leslie matrix is indeed independent of time. We thus decided to compute a single curve for $P(x)$ by pooling all the cells together independently of the time at which they were measured. The resulting curve shows that the survival probability becomes negligible after 150 minutes, with a medium age at division of 80 minutes.

We found that the exponential growth rate of the population, Eq (29), is $\rho \sim 0.0089/min$, which is the same as the measured single cell growth rate. As expected, the population age structure differs markedly between the observed and the

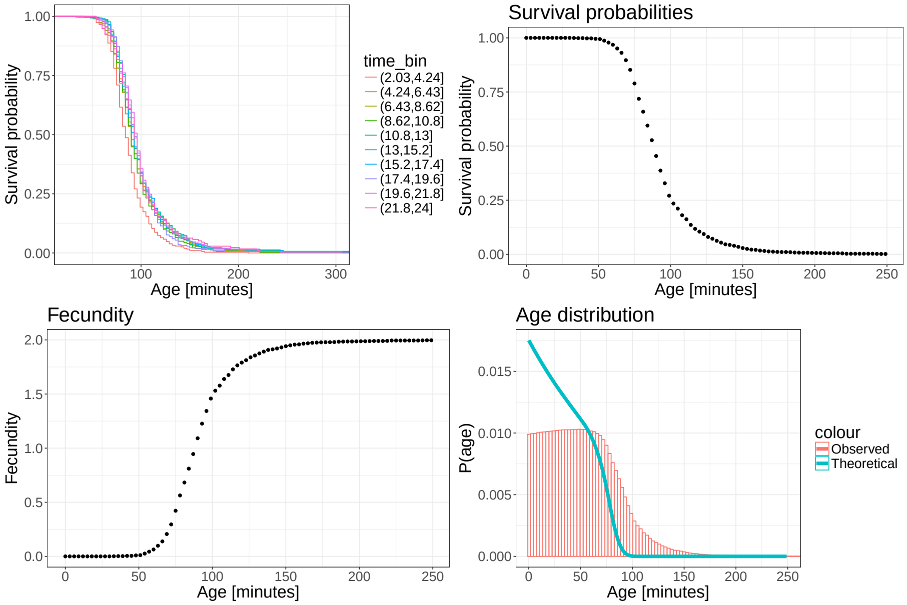

Fig S8. The Leslie model. *Top left*: The one-time point survival probabilities don't depend on the time and they show that the survival probability becomes negligible for cells older than about 150 minutes. The medium age at division is around 80 minutes. *Top right*: Since the probabilities don't depend on time, we pooled data from all times together to obtain a single survival curve. *Bottom left*: Fecundity as function of cell age. *Bottom right* Asymptotic age distribution of the population for large times as observed (red) and predicted by the theory (blue). Note that the observed distribution has an over-representation of old cells relative to the theoretically predicted distribution.

theoretical one, as shown on the bottom right part of Fig S8. In particular the theoretical distribution predicts more young cells than old, while in the microfluidic device we have a nearly uniform age distribution with a right tail; hence, as expected, young cells are relatively under-represented in the population in the microfluidic device.

Assuming that the GFP concentration is constant, the total GFP must depend on the cell age through the length and in computing the mean and variances we have to weight the GFP by the ratio between the theoretical and the observed age fractions. Specifically, if $P_{obs}(\text{age})$ and $P_{th}(\text{age})$ are the observed and the theoretical age distributions, then for the $\log(GFP)$ distribution it holds

$$\bar{g} = \frac{\sum_{i=1}^{N} g_i\,\omega(\text{age}_i)}{\sum_{i=1}^{N}\omega(\text{age}_i)} \qquad (30)$$

$$\text{Var } g = \frac{\sum_{i=1}^{N}(g_i-\bar{g})^2\,\omega(\text{age}_i)}{\sum_{i=1}^{N}\omega(\text{age}_i)} \qquad (31)$$

where i runs over all cell observations, $g \equiv \log(G)$ and $\omega(\text{age})$ is a weighting factor that accounts for the over-representation of older cells

$$\omega(\text{age}) = \frac{P_{th}(\text{age})}{P_{obs}(\text{age})} \qquad (32)$$

Going into log-space, we have estimates more robust against outliers and we can approximate the CV^2 in the real space as the variance in the log-space. Suppl. Fig. S9 shows that the age structure correction makes the CVs only slightly lower than the one measured ignoring the age structure effects.

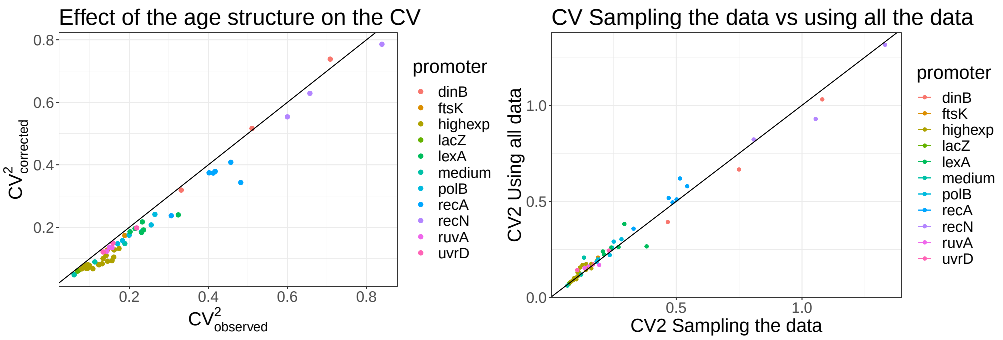

Fig S9. Effect of the age structure on the measured CV^2. *Left*: CV^2 corrected by the age structure in the microfluidic device versus the CV^2 as directly measured, ignoring the age structure effect. The age structure has the effect of making the measured CV^2 slightly higher than the CV^2 corrected by the age structure of the population. *Right*: CV^2 obtained by taking all the measures in the microfluidic device versus the CV^2 when taking only one point, chosen at random, per cell. We can see that the difference is very small and the points accumulate around the bisector.

6.2 Correlation in the measurements

As we are recording the size and GFP expression of cells every 3 minutes in the microfluidic device, observations from nearby time points are clearly correlated, and it could be suspected that this may affect the statistics we calculate. In contrast, in the FCM, we have cells measured at only one time point, so we don't have correlations coming from the fact that we have multiple data points coming from the same cell. To

check whether the presence of these correlated data points can substantially bias the statistics, we measured the CV^2 for different promoters and media on different dates in two different ways:

1. Taking all the data, regardless of the fact that they may come from the same cell.
2. Taking only one randomly selected data point for each cell.

The first condition is the one that we use throughout the paper to compute the statistics, while the second one should be closer to the FCM setup. Suppl. Fig. S9 shows that the CV^2 are almost identical whether correlated time points are included or not. In conclusion, neither the age structure nor the correlated measurements affect the comparison of CV^2 between measurements from the FCM and from the microfluidic device.

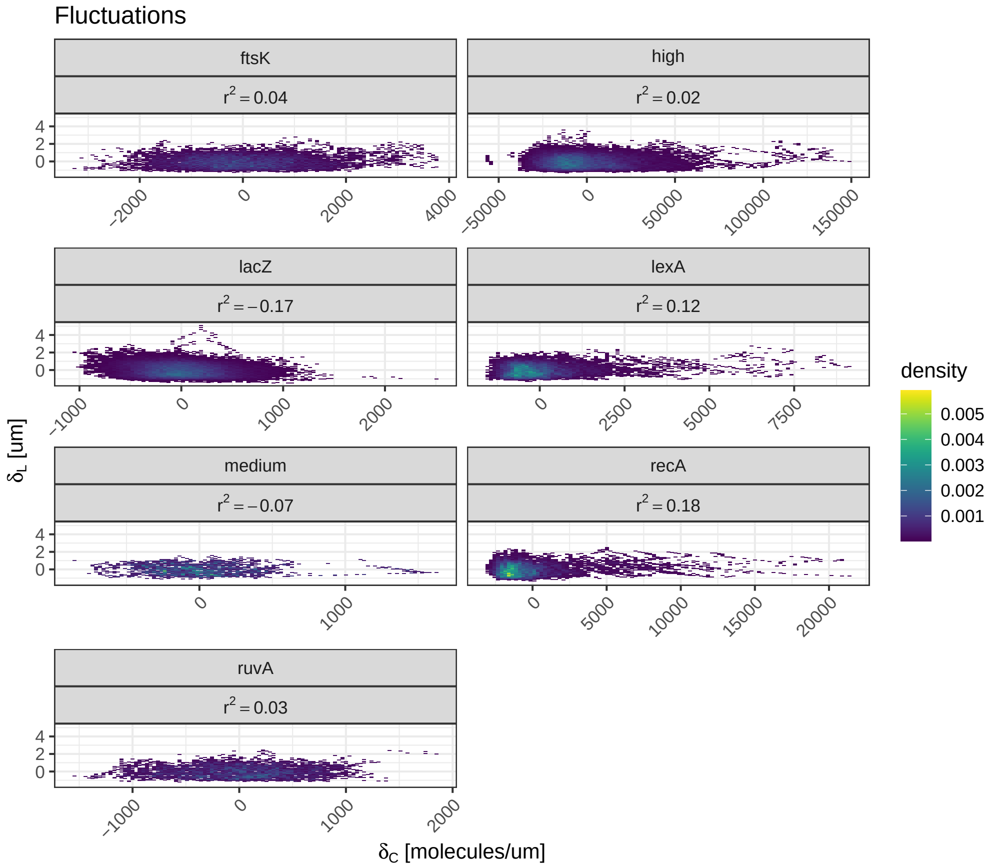

Fig S10. Fluctuations in size are independent on fluctuations on concentration. The fluctuations in length ($\delta_L = L - \langle L \rangle$) are independent of the fluctuations in GFP concentration (δ_C). Each subpanel is a different promoter measured in the mother machine setup and the squared Pearson correlation coefficient is shown under the promoter name.

References

1. Biosciences B. BD FACSCanto II Flow Cytometer Reference Manual; 2006.

2. Verwer B. BD FACSDiVa Option. White Paper;. http://www.bdbiosciences.com/ds/is/others/23-6579.pdf.

3. Zaslaver A, Bren A, Ronen M, Itzkovitz S, Kikoin I, Shavit S, et al. A comprehensive library of fluorescent transcriptional reporters for Escherichia coli. Nature Methods. 2006;3.

4. Urchueguía A, Galbusera L, Bellement G, Julou T, van Nimwegen E. Noise propagation shapes condition-dependent gene expression noise in Escherichia coli. BioRxiv. 2019;doi:10.1101/795369.

5. Balwierz PJ, Pachkov M, Arnold P, Gruber AJ, Zavolan M, van Nimwegen E. ISMARA: automated modeling of genomic signals as a democracy of regulatory motifs. Genome Research. 2014;24(5):869–884. doi:10.1101/gr.169508.113.

6. Eberhardt EH. Noise in photmultiplier tubes. IEEE. 1967;.

7. Charlesworth B. Evolution in age-structured populations. 2nd ed. Cambridge University Press; 1994.
