# Supplementary material for: Using fluorescence flow cytometry data for single-cell gene expression analysis in bacteria
Source: PLoS One. 2020 Oct 12;15(10):e0240233. doi: 10.1371/journal.pone.0240233 (PMC7549788; doi:10.1371/journal.pone.0240233)
Supplement: Attachment — Submitted filename: rebuttal_reviews.pdf [file pone.0240233.s002.pdf]

Response to the reviews of PONE-D-19-27976: Using fluorescence flow cytometry data for single-cell gene expression analysis in bacteria

Flow cytometry has many applications and methods for processing of flow cytomeytry data of course strongly depend on the application. Our paper concerns processing of data for the specific application of measuring single-cell gene expression in bacteria using fluorescent reporters. Although this is certainly not the most common application of flow cytometry, this particular subfield has by now become well established and there is a substantial number of works in well-regarded journals in which this methodology has been used, e.g. [1–22]. Our work is about processing of flow cytometry data from this specific application.

Since the paper is mainly targeted toward researchers that use flow cytometry for this application, it was written under the assumption that the reader is familiar with this application. We now understand it was a mistake to assume this and have addressed this in the revision. We have also added new results to address the main concern of reviewer #1 (non-linearity) and the main concern of reviewer #2 (that our results are not at odds with previous literature on using flow cytometry to estimate cell sizes in prokaryotes).

Reviewer #1

Reviewer #1 starts by summarizing our work as: *"The authors aim to correct flow cytometric measurements of GFP expression in E.coli for non-linearity in order to arrive at better estimates of GFP expression."* This is simply not true. Our work is not at all about correcting for non-linearity. Although we do not doubt that non-linearity can be an issue in flow cytometry, for our application non-linearity between microscope and FACS measurements is not a problem at all. How the reviewer has gotten the idea that this is the topic of our work is a complete mystery to us because the phrase 'non-linearity' does not even appear anywhere in the paper. Therefore, the following claims by this reviewer are all false and based on a fundamental misunderstanding of what our paper is about:

- *The premise of the experiments is that a numeric process for linearizing the data obtained will apply to other flow cytometry instruments with different optics, illumination conditions, alignment, and sample preparation techniques.*
- *Instead they compare microscope and flow cytometry measurements made under arbitrary conditions and present a numerical method to linearize the two.*
- *The linearization of the measurements may give satisfactory results in the author's lab (although, given the fact that they do not present alignment procedures this is by no means certain and that can expect large day-t-day variability), it is doubtful that their procedure would be helpful in comparing results between different labs particularly if such labs would imply different instruments.*

Although all these remarks are thus based on faulty assumptions, for the revision we have nonetheless set out to explicitly show that non-linearity is not an issue at all for our application. In particular, we show that the fluorescence measurements of FACS and microscope are in fact linear over the entire range of gene expression levels. This should also address all of this reviewer's general concerns about non-linearity, including:

- *Fluorescence is intrinsically a non-linear process. Flow cytometric fluorescence measurements of small particles are not necessarily proportional to the content of fluorescent analyte contained in each particle.*

- *Fluorescence measurements are subject to molecule- density dependent energy transfer. Transfer processes (non-linearly) affect fluorescence efficiency, color and polarization.*

- *Given the intrinsic non-linearity of fluorescence and the difference in behavior of small versus larger particles, the non-proportionality with fluorescence emission and chromophore content, complications caused by polarization effects and the complex shape and non-homogeneity of microbes, an article on the standardization of fluorescence results should start with a careful analysis and description of the instrument and alignment criteria.*

Beyond this, the reviewer makes a number of remarks about basic issues that are of concern when designing a flow cytometer. These include:

- *The authors do not describe details of the optical geometry nor the method by which the electronics that translate fluorescences pulses into digital values. They do not describe their alignment procedures.*

- *Light scatter is highly dependent on measurement angle and polarization sensitivity of the detectors.*

- *an article on the standardization of fluorescence results should start with a careful analysis and description of the instrument and alignment criteria.*

- *The fail to indicate the angular distribution of the scatter of small particles (Mie versus classical scatter).*

- *Calibration of measurements needs to start with the calibration of instruments and methods.*

We are not designing a flow cytometer or proposing new measurement procedures. We are using a standard commercial FACS machine in a standard manner, as has been done already in many publications, i.e. to measure gene expression in single bacterial cells using fluorescent reporters [1–22]. We are not introducing or proposing new measurement procedures. Our paper is solely concerned with *data analysis* procedures. These remarks of the reviewer are thus also not relevant.

Finally, the reviewer makes a number of statements that are factually incorrect:

- *They do not present efforts to standardize their samples.* In fact the data we analyse were obtained using standard protocols used by researchers in this field.

- *They fail to indicate that GFP takes several hours to fold into a configuration with a constant fluorescence efficiency.* In fact the GFP used in this study has been demonstrated to mature in under 10 minutes [23].

- *They fail to indicate what measures were taken to prevent photobleaching and photon saturation.* In fact photobleaching does not meaningfully affect the measurements because each cell is only illuminated once. Regarding saturation, it can be clearly seen from the raw fluorescence distributions shown in several of our figures that there is no saturation at high intensities.

- *The linearization of the measurements may give satisfactory results in the author's lab (although, given the fact that they do not present alignment procedures this is by no means certain and that can expect large day-t-day variability).* In fact the paper show results from different days in many places and uses them to calculate error bars on estimates.

We understand that, because our paper was written assuming familiarity with the literature on using flow cytometry for single-cell gene expression measurement in bacteria, this may have led to some confusion. In the revision we have made sure to introduce the subject and the aims of our analysis procedures more clearly for researchers that are not familiar with this area.

However, although we take some responsibility for confusing the reviewer, we do take issue with the strong and absolute hostility of this review. We really see no evidence at all that the reviewer has made a fair attempt at trying to understand what was done in our paper.

Reviewer #2

The criticisms of reviewer #2 all result from the lack of familiarity of this reviewer with the application of flow cytometry to estimate single-cell gene expression in bacteria, leading to the following three misunderstandings:

1. That we are developing this method in this paper.
2. That there is no connection between the estimation of cell size and gene expression.
3. That our claims that cell size cannot be reliably estimated using flow cytometry is at odds with existing literature.

Regarding point 1, as the citations above show, flow cytometry has been used to estimate single-cell expression in bacteria for more than 20 years, and high-throughput applications have existed for a decade.

Regarding misunderstanding number 2, single-cell gene expression in bacteria is most commonly measured using microscopy, i.e. by quantifying the GFP *concentration* in each cell. Here we are investigating to what extent it is possible to estimate the GFP concentration of single cells using flow cytometry as well. This requires that we estimate, for each cell, both its size and its GFP content from the fluorescence and forward/side-scatter and measurements on that single cell. That is, we are concerned with populations of cells of a single strain growing in a homogeneous environment and investigate to what extent the subtle differences in the sizes of single cells in this population can be estimated from single forward/side-scatter measurements. Our results show that flow cytometry measurements of single cells are too noisy to meaningfully do this.

Misunderstanding number 3 results from confusing this problem of estimating sizes of *single* cells from *single* scattering measurements from the problem of estimating *average* sizes of populations of cells from the averages of *many* scattering measurements. The reviewer is clearly aware of this latter application, e.g. to use flow cytometry to distinguish different bacterial species of different shapes and sizes, but fails to distinguish it from the much subtler problem of estimating variations in single-cell sizes from individual scattering measurements. Virtually all criticism of this reviewer stems from this misunderstanding. Our results are not at all at odds with this existing literature, which we now explicitly demonstrate in the revision as explained in the next parapgraph.

In retrospect we realize that, because our paper was written under the assumption that readers are familiar with the application of flow cytometry to estimating single-cell gene expression in bacteria, the presentation was confusing to readers that are completely unfamiliar with this application. We can thus understand reviewer #2's confusion and believe that this can be easily cleared up. In the revision we have not only introduced this topic much more clearly and explicitly, we also clearly explain the difference between our cell size estimation problem (i.e. estimating variations in sizes of single cells) and the problem of estimating population averages. In addition, we now include a new section of results where we show that, consistent with existing

literature that the reviewer cites, we can also successfully use averages of forward/side-scatter to estimate the differences in average sizes of populations of cells growing in different media. We think this should clear up essentially all criticisms that reviewer #2 had.

More Detailed responses to the comments of reviewer #2

Comment 1: *The study by Galbusera et al investigates the viability of flow cytometry as a tool for measuring characteristics of individual cells, including cell size and the expression of specific genes. The paper is timely as the field moves towards functionally driven single cell-omics approaches and expression analyses and robust validation of commonly used techniques is necessary and crucial for future advancements.*

Please note that while I am happy to review the biological aspects of this study, I do not feel qualified to comment on the mathematical methods employed within the E-Flow R package to calibrate FACS measurements.

Response 1: We thank the reviewer for being upfront about this. However, since our paper is mainly about data processing methods, this absence of quantitative expertise does make it hard for the reviewer to judge the validity of our work.

Comment 2: *I have multiple serious concerns about the conclusions that the authors draw about the viability of flow cytometry as a valid technique for the calculation of cell size in prokaryotic. First, this study directly contradicts multiple previous studies (DOI 10.1002/cyto.990100112, DOI 10.1038/s41467-017-00128-z, and PMID 9758817 for example) that demonstrated that flow cytometry can be used to estimate cell size of prokaryotic cells using transformations of forward scatter, and this disparity of results needs to be directly addressed. I would recommend that the authors revisit the literature and investigate some of the techniques that have previously been reported and see if those techniques would address the lack of correlation they report, before they declare the technique invalid.*

Response 2: This is a complete misunderstanding, probably resulting from the reviewer being unfamiliar with the application of flow cytometry that we study, i.e. estimating single-cell gene expression in bacteria. Importantly, there is no contradiction at all. The studies the reviewer mentions concern distinguishing the *average* size of different types of prokaryotic cells by taking many measurements of the cells of each type, and calculating averages of the forward and side-scattering of each type. We completely agree that prokaryotes that have sufficiently different shape and size can be distinguished using such measurements.

In contrast, we are considering populations of cells of a single bacterial strain growing exponentially in homogeneous conditions, and we are asking whether it is possible to distinguish the small variations in size of single cells using single forward- and side-scatter measurements. In particular, we are asking whether forward/side-scatter measurements on single cells can estimate cell size accurately enough to be able to meaningfully estimate GFP *concentrations* rather than just total GFP levels. The results in our paper show that this is impossible.

In the revision we have rewritten the corresponding sections to clearly distinguish these two problems. In addition, we have added an entirely new analysis section (Fig. 3 of the revised manuscript) to confirm, in correspondence with existing literature that the reviewer cites, that it is indeed possible to use averages of forward- and side-scatter measurments to estimate changes in average size of bacterial cells. In particular, it is well-known that E. coli's average cell size increases with growth-rate. We use measurements of E. coli cells growing in different growth media (for which growth rates vary by roughly 6-fold) and comparison of average forward/side-scatter measurements with size estimates from microscopy experiments to show that FACS measurements can indeed estimate changes in average size of cells with reasonable accuracy. We think that these new results should clear up this misunderstanding.

Comment 3: *As the authors allude to rod shaped bacteria can pass through the detector in a variety of confirmations that could easily account for a significant amount of the variation that they observe in the forward and side scatter measurements. I would encourage the authors to revisit their calculations and the available literature. Secondly, bacteria have a tremendous variety of shapes and sizes that they can be and this study only examined a single species and a single shape. It would greatly benefit the study and strengthen the authors conclusions if this study were repeated on multiple other shapes including another rod, 2 cocci and ideally a spirillum, and produced consistent results across morphologies. I am extremely hesitant to believe any study that claims a technique is (in)valid for all bacteria (and archaea) based on the results from a single species. This is hesitancy is heightened by one of the previous studies already mentioned that did examine multiple species and shapes and found that forward scatter can be used to estimate cell size.*

Response 3: We feel that these comments have now become irrelevant since they were based on a misunderstanding of what we were claiming. We fully agree average sizes can be estimated using FACS measurements.

Comment 4: *Finally, while the development of a method to quantify expression data in a high-throughput method is a fundamental advancement that I believe is of great importance to the field the results presented here need some additional data to support them. Again, I would have liked to have seen the method applied to additional cell types to provide evidence of the applicability of the methodology to a range of cells beyond just E. coli.*

Response 4: The fact that the reviewer seems to think that we are 'developing' the application of flow cytometry to measure gene expression in single cells indicates that the reviewer is unaware that this method has already been applied in numerous works over two decades, e.g. [1–22]. Our paper is concerned with methods for *processing data* from such measurements and shows that, in order for single-cell expression distributions measured in FCM and using microscopy to match, one has to properly account for different levels of autofluorescence, and correct for shot-noise that is introduced by the FCM measurements.

Although it would be interesting to analyze other types of bacteria, we feel that this is beyond the scope of this paper, which is mainly concerned with data analysis procedures and not obtaining new datasets. First, almost all existing studies of single-cell gene expression using flow cytometry that we are aware of involve rod-shaped bacteria (mostly E coli). Second, although the shape of bacterial cells will likely affect the characteristics of forward/side-scatter measurements, cell shape is unlikely to affect the characteristics of the total fluorescence measurements. Thus, our primary analysis methods for processing fluorescence measurements are unlikely to change for bacteria of different shape.

Comment 5:*I would recommend that the authors either, reexamine the relationship between scatter and cell size of E. coli and other microbial species and potentially between cell size and gene expression or that they separate the second part of the manuscript assess the expression technique on 1-3 additional species. As it is currently written I feel as if the manuscript is two independent experiments that they attempted to stick together to make a full-length manuscript.*

Response 5: This comment shows that the reviewer has not understood that our cell size analysis is specifically connected to the gene expression analysis. In particular, because GFP levels increase with cell size, one would ideally estimate GFP concentrations rather than just total GFP levels. As we show in the paper, for microscope measurements this is possible and the variability in inferred GFP concentrations across cells is significantly smaller than the variance in total GFP levels, i.e. a considerable amount of the variance in total GFP is explained by variation in cell size.

For the flow cytometry data, one would similarly like to estimate GFP concentrations for single cells by estimating cell size from the single-cell forward/side-scatter measurement, and

then divide the total fluorescence by this estimated size. In our paper we show that the cell-size estimates based on forward/side-scatter are too noisy to make such an approach viable.

References

1. Chung JD, Stephanopoulos G, K I, D GA. Gene expression in single cells of Bacillus subtilis: evidence that a threshold mechanism controls the initiation of sporulation. Journal of bacteriology. 1994;176. doi:10.1128/jb.176.7.1977-1984.1994.

2. Valdivia RH, Falkow S. Bacterial genetics by flow cytometry: rapid isolation of Salmonella typhimurium acid-inducible promoters by differential fluorescence induction. Mol Microbiol. 1996;22(2):367–378.

3. Wilson RL, Tvinnereim AR, Jones BD, Harty JT. Identification of Listeria monocytogenes in vivo-induced genes by fluorescence-activated cell sorting. Infect Immun. 2001;69(8):5016–5024.

4. Ozbudak EM, Thattai M, Kurtser I, Grossman AD, van Oudenaarden A. Regulation of noise in the expression of a single gene. Nat Genet. 2002;31(1):69–73.

5. Hakkila K, Maksimow M, Rosengren A, Karp M, Virta M. Monitoring promoter activity in a single bacterial cell by using green and red fluorescent proteins. J Microbiol Methods. 2003;54(1):75–79.

6. Sevastsyanovich Y, Alfasi S, Overton T, Hall R, Jones J, Hewitt C, et al. Exploitation of GFP fusion proteins and stress avoidance as a generic strategy for the production of high-quality recombinant proteins. FEMS Microbiology Letters. 2009;299(1):86–94. doi:10.1111/j.1574-6968.2009.01738.x.

7. Miao H, Ratnasingam S, Pu CS, Desai MM, Sze CC. Dual fluorescence system for flow cytometric analysis of Escherichia coli transcriptional response in multi-species context. J Microbiol Methods. 2009;76(2):109–119.

8. Kinney JB, Murugan A, Callan CG, Cox EC. Using deep sequencing to characterize the biophysical mechanism of a transcriptional regulatory sequence. Proc Natl Acad Sci USA. 2010;107(20):9158–9163.

9. Anand R, Rai N, Thattai M. Promoter reliability in modular transcriptional networks. Meth Enzymol. 2011;497:31–49.

10. Silander OK, Nikolic N, Zaslaver A, Bren A, Kikoin I, Alon U, et al. A Genome-Wide Analysis of Promoter-Mediated Phenotypic Noise in Escherichia coli. PLOS Genetics. 2012;8(1):1–13. doi:10.1371/journal.pgen.1002443.

11. Madar D, Dekel E, Bren A, Zimmer A, Porat Z, Alon U. Promoter activity dynamics in the lag phase of Escherichia coli. BMC Syst Biol. 2013;7:136.

12. Sanchez-Romero MA, Casadesus J. Contribution of phenotypic heterogeneity to adaptive antibiotic resistance. Proc Natl Acad Sci USA. 2014;111(1):355–360.

13. Utratna M, Cosgrave E, Baustian C, Ceredig RH, O'Byrne CP. Effects of growth phase and temperature on IfB activity within a Listeria monocytogenes population: evidence for RsbV-independent activation of IfB at refrigeration temperatures. Biomed Res Int. 2014;2014:641647.

14. Wolf L, Silander OK, van Nimwegen E. Expression noise facilitates the evolution of gene regulation. eLife. 2015;4:e05856. doi:10.7554/eLife.05856.

15. Baert J, Kinet R, Brognaux A, Delepierre A, Telek S, S?rensen SJ, et al. Phenotypic variability in bioprocessing conditions can be tracked on the basis of on-line flow cytometry and fits to a scaling law. Biotechnol J. 2015;10(8):1316–1325.

16. Yan Q, Fong SS. Study of in vitro transcriptional binding effects and noise using constitutive promoters combined with UP element sequences in Escherichia coli. J Biol Eng. 2017;11:33.

17. Nordholt N, van Heerden J, Kort R, Bruggeman FJ. Effects of growth rate and promoter activity on single-cell protein expression. Sci Rep. 2017;7(1):6299.

18. Rohlhill J, Sandoval NR, Papoutsakis ET. Sort-Seq Approach to Engineering a Formaldehyde-Inducible Promoter for Dynamically Regulated Escherichia coli Growth on Methanol. ACS Synth Biol. 2017;6(8):1584–1595.

19. Razo-Mejia M, Barnes SL, Belliveau NM, Chure G, Einav T, Lewis M, et al. Tuning Transcriptional Regulation through Signaling: A Predictive Theory of Allosteric Induction. Cell Syst. 2018;6(4):456–469.

20. Belliveau NM, Barnes SL, Ireland WT, Jones DL, Sweredoski MJ, Moradian A, et al. Systematic approach for dissecting the molecular mechanisms of transcriptional regulation in bacteria. Proc Natl Acad Sci USA. 2018;115(21):E4796–E4805.

21. Bahrudeen MNM, Chauhan V, Palma CSD, Oliveira SMD, Kandavalli VK, Ribeiro AS. Estimating RNA numbers in single cells by RNA fluorescent tagging and flow cytometry. J Microbiol Methods. 2019;166:105745.

22. Urchueguía A, Galbusera L, Bellement G, Julou T, van Nimwegen E. Noise propagation shapes condition-dependent gene expression noise in Escherichia coli. BioRxiv. 2019;doi:10.1101/795369.

23. Balleza E, Kim JM, Cluzel P. Systematic characterization of maturation time of fluorescent proteins in living cells. Nat Methods. 2018;15(1):47–51.
