# Supplementary material for: Using fluorescence flow cytometry data for single-cell gene expression analysis in bacteria
Source: PLoS One. 2020 Oct 12;15(10):e0240233. doi: 10.1371/journal.pone.0240233 (PMC7549788; doi:10.1371/journal.pone.0240233)
Supplement: Attachment — Submitted filename: responses_to_reviews.pdf [file pone.0240233.s003.pdf]

Response to the reviews of PONE-D-20-01335: Using fluorescence flow cytometry data for single-cell gene expression analysis in bacteria

Reviewer #1

Comment 1: *Galbusera et al, presented a study on the analysis and use of fluorescence flow cytometry data, to study single-cell of bacteria that express a reporter gene at different concentrations. The work is complete and data are sufficient for pubblication. The authors also provide a tool for the measurements of autofluorescence and shot noise of the instrument. However, these results highlight the fact that this technique is not very efficient in the single cell study but rather for the evaluation of the fluorescence signals of an entire bacterial population. The title may mislead the reader who expects to find a single cell analysis approach. I suggest to clarify this point in the title or in the main text.*

Response 1: This is a misunderstanding. Our study does very much concern the single-cell analysis of gene expression using fluorescence flow cytometry. That is, the flow cytometer provides independent fluorescence and scattering measurements for every single cell. Like any measurement technology, these measurements are subject to measurement noise. It happens to be the case, as we show in our manuscript, that for isogenic populations of bacterial cells growing in a homogeneous environment, the size of this measurement noise is on the same order of magnitude as the true variability in GFP fluorescence across cells. Consequently, the accuracy of the estimates of total GFP in single cells are limited. However, we show that even though individual measurements are highly noisy, it is still possible to accurately estimate the true variability in GFP across cells, and develop methods to do so. We regret that this apparently created the impression that we claim flow cytometry can only be effectively used for bulk population measurements. Notably, quantifying the true variance in GFP levels across single cells very much requires single cell measurements and cannot be estimated from measurements on bulk populations.

To avoid this misunderstanding, we have made alterations to the abstract and the main text to make these points clearer.

Reviewer #2

Comment 1: *Choice of signal parameters to monitor: pulse width is typically used to discriminate single particles from doublets. I am therefore not surprised that this parameter is not particularly informative for fluorescence quantification. It is reassuring that the area is proportional to the product of pulse height and width for the calibration beads. Does a similar connection hold for bacterial cells as well?*

Response 1: The fact that the area is proportional to the product of height and width of the signal appears to be a generic property of the electronics that the flow cytomer uses to compute the signals, and it also applies to bacterial cells (as shown in supplementary Fig. S1).

Comment 2: *Gating events based on the scatter measurements: in various ways, this is routinely done by several groups that practice flow cytometry, either in a manual or semi-automatic manner. Does the approach proposed by the authors have any significant advantages?*

Response 2:

The advantage of our approach is that our gating strategy is based on a careful analysis of each signal reported by the fluocytometer. Therefore, we believe it is a more systematic and principled way of filtering out debris, compared to the plethora of often rather *ad hoc* methods found in the literature. Second, whereas it is sometimes assumed that a tight gating strategy

can effectively select out bacterial cells of similar size, our analysis shows that this is not that case, i.e. the distributions of total fluorescence are almost indistinguishable whether one uses very tight or lenient gating. Our analysis thus also makes clear that there is no advantage to aggressively filter events.

We discussed the advantages of our method in the disscussion section, but we agree with the reviewer that it is helpful to already do so earlier in the text. We now also discuss them in the introduction.

Comment 3: *Correlation of FSC and SSC with mean cell size: here, the authors are missing an important citation (Volkmer & Heinemann, PLoS ONE, 2011). That work also made a comparison between mean FSC/SSC and cell volume, with very similar conclusions to the ones made here.*

Response 3: We agree and have now included this highly relevant reference. We thank the reviewer for pointing out this oversight.

Comment 4: *Use of FSC/SSC to measure individual cell sizes: the authors conclude that FSC/SSC signals do not carry enough information to measure cell size distributions in an E. coli population. Though I would tend to agree with this conclusion, I do not fully agree with the arguments provided. The authors apply equations (1) and (2) both to microscopy and flow cytometry data. While it is clear to me that (1) and (2) are perfectly adequate for size measurements based on microscopy, I am not sure that they apply just as easily in the case of flow cytometry. Specifically, eq. (1) could perhaps look like* $x_m = f(x_t) + \epsilon$*, i.e. the relationship between measured and actual sizes could be nonlinear at worst, or linear (perhaps with an offset) at best. In either case, the variance of* x_m *for flow cytometry will not obey (2), even in the simplest scenario of linear scaling without offset. I think that, before attempting to decompose the measured variance of the flow cytometry signals, the authors should first infer the relationship between* x_m *and* x_t *in the case of flow cytometry, for example by using calibration beads of different sizes. Then, using variance propagation they could estimate* $var(x_t)$ *based on* $var(x_m)$ *and determine how much information the FSC/SSC signals carry about the size of single cells. Another point regarding the analysis of this section is that one should probably also suspect the noise in GFP measurements as another source of error. As the authors show later on, the GFP signal requires some significant corrections to correctly report the variability of the cell population. On the other hand, it seems to me that the GFP signals used in Fig. 4 were not corrected for shot noise and autofluorescence. It is therefore plausible that the lack of correlation between GFP and FSC/SSC can be partially attributed to the GFP noise.*

Response 4: Although we are still convinced (like the reviewer) that FCM scattering measurements are too noisy to usefully estimate sizes of individual cells, we agree with the reviewer that our simple analysis quantifying the FCM measurement noise made unwarranted assumptions. We investigated whether there was a straightforward way to correct our method for estimating the accuracy of the cell size estimates, but eventually decided that there are in the end too many unknowns (i.e. both the unknown non-linear dependence of scattering on true cell size, and the unknown scaling of the noise with the size of the signal) to be able to do this without leaving any loopholes open. Although we understand the reviewer's suggestion to use synthetic beads for this task as well, the relationship between scatter and true size depend on the material and shape of the particles, so that the measurements on the synthetic beads are not informative for the behavior with bacterial cells.

Since the main purpose of estimating sizes of single cells is to be able to measure the distribution of GFP *concentrations* across cells, we have in the revision completely rewritten this section of the paper to focus on the question of estimating concentration fluctuations. In this new section we first show that, whereas the average scatter of a population of cells can be used to estimate the average size of the cells, there are different non-linear relationships between the

different scattering signals and cell size, and these relationships are likely specific to our specific setup. In addition, by comparing coefficients of variation of cell size as measured by microscopy with coefficients of variation of the scattering signals, we show that the the scattering signals also contain a large shot noise, whose size cannot be easily calibrated. Together, these observations argue that there is no straightforward way to accurately estimate GFP concentrations in single cells. However, we also show that, because GFP concentrations and cell sizes fluctuate roughly independently, the relative size of GFP concentration fluctuations for different genes can still be reasonably estimated.

Finally, we agree with the reviewer that the absence of correlation between the scattering and fluorescence signals in Fig. 4 results both from the shot noise on the GFP measurements as well as the noise on cell size measurements. We have made this clear in the revision.

Comments 5&6: *The authors perform an analysis which is typically carried out in one or another way by practioners of flow cytometry. However, their approach looks quite principled and systematic (e.g. in the averaging of autofluorescence measurements from different dates).*

Response 5&6: We thank the reviewer for this comment.

Comment 7: *Calibration of fluorescence signal to remove shot noise and autofluorescence: here, the authors perform a nice analysis to remove the additional variability introduced by flow cytometry using calibration beads (an approach similar to what I suggested above for the FSC/SCC analysis), and are eventually able to achieve relatively good agreement between microscopy and flow cytometry measurements. I would have preferred to also see the relative, besides the absolute error in the CV's, to better understand over which range of GFP concentrations the flow cytometry measurements can provide a reliable estimate of the GFP variability.*

Response 7: We agree that we should have also provided a plot directly comparing the final CV^2 of each gene as estimated by our procedure for the FCM data, with the corresponding CV^2 as estimated from the microscopy data. In the revision we now provide such a plot (Fig. 8) showing that, only after correcting for fluorescence and shot noise is there a good correlation between the CV^2 as estimated by these two measurement technologies.

Other comments

1. *Strains and growth conditions section (M&M) needs some streamlining to better organize the information. Growth media, precultures and dilutions are a bit mixed up for flow cytometry and microscopy experiments, and a bit hard to follow.*

   We have rewritten this section to clearly separate the FCM from the microscopy experiments.

2. *Fig. 3: would it be possible that the authors repeat some of the information of ref. 31 about the medium conditions? For example, what does "salt" mean? How are cells grown in these different media? (is growth balanced, for example?)*

   We added a paragraph in the *Materials and Methods - Strains and growth condition* and the new section *Materials and Methods - Size estimation* which we think provides sufficient information on the different growth conditions. For more details we refer the reader to the original study.

3. *Fig. 4: what are the units of the horizontal axis in the second and third rows? The scattering units used here are different from those of other figures where scatters are displayed.*

   That was a typo left over from a previous version of the plot. We corrected the units and thank the reviewer for catching this error.

4. *Fig. 5: is it correct that the two strains mentioned here are simply the same background strain carrying two different plasmids?*

Yes, they are the same strains carrying two different plasmids.

5. *Fig. S6: I do not understand the x-axis title (mean log (GFP.H)).*

   This was also an error. We meant log(GFP.H) and not the mean of it. We fixed the label and thank the reviewer again for catching the mistake.
